# Corrosion Behaviour in CO_2_ Pipeline Transport: A Review of the Impact of Condensates and Impurities

**DOI:** 10.3390/ma19102048

**Published:** 2026-05-14

**Authors:** Luca Gritti, Denny Coffetti, Lorenzo Nani, Sergio Lorenzi, Marina Cabrini

**Affiliations:** 1 Department of Engineering Applied Science, University of Bergamo, 24044 Dalmine, Italy; denny.coffetti@unibg.it (D.C.); lorenzo.nani@unibg.it (L.N.); sergio.lorenzi@unibg.it (S.L.); marina.cabrini@unibg.it (M.C.); 2 INSTM, Consorzio Interuniversitario Nazionale per la Scienza e Tecnologia dei Materiali, 50121 Firenze, Italy; 3 CSGI, Consorzio Interuniversitario per lo sviluppo dei Sistemi a Grande Interfase Center for Colloid and Surface Science, 50019 Sesto Fiorentino, Italy

**Keywords:** energy transition, CCTS, impurities, corrosion rate, top of the line corrosion

## Abstract

**Highlights:**

- Role of the principal CCTS strategy and effect on the resulting flux
- The role of water in the CO_2_ flux and Top of the line corrosion
- Role of the different impurities on the corrosion behaviour

**Abstract:**

The high emissions of carbon dioxide (CO_2_) into the atmosphere have driven the development of carbon capture, transport, and storage (CCTS) technologies. These focus on capturing CO_2_ from industrial exhaust gases and transporting it through existing pipeline networks. Although various capture techniques are available, they may introduce impurities such as O_2_, N_2_, Ar, H_2_O, NH_3_, and others into the CO_2_ stream. These contaminants can significantly alter the thermophysical behaviour of the fluid, making the phase behaviour predictions, reliable for pure CO_2_, much more complex. Pressure and temperature variations along pipelines can induce unexpected phase transitions, affecting fluid composition and potentially triggering corrosion. This review examines the formation of condensates within pipelines and their role in initiating corrosion phenomena, with a focus on top of the line corrosion (TLC) and conventional CO_2_-induced corrosion (sweet corrosion). The main literature findings highlight how phase changes and altered fluid composition due to corrosion processes can significantly intensify degradation mechanisms during CO_2_ transport.

## 1. Introduction

### 1.1. Energy Transition: The CO_2_ Impact

**Figure 1:**
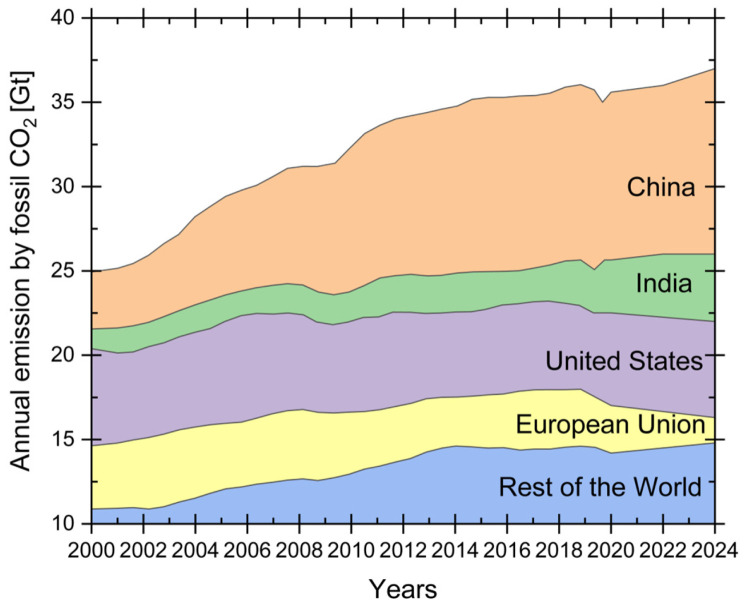
Global CO_2_ emissions from fossil fuels by regions, 2000–2024, adapted from [5], showing emissions from the major countries and global regions.

The continuous growth in global energy demand, coupled with the worsening of environmental issues, has led to the establishment of international agreements aimed at limiting and reducing carbon dioxide (CO_2_) emissions into the atmosphere. The United Nations Paris Agreement set the ambitious goal of controlling the rise in the average global surface temperature of the Earth [1]. To achieve this target, global CO_2_ emissions (GCE) must be reduced by 50–85% compared to the emission levels of the year 2000 [2,3] in order to counteract the opposing international trend shown in Figure 1 [4,5]. One of the promising and implementable strategies is the set of technologies known as carbon capture, transport, and storage (CCTS). The aim is to capture carbon dioxide directly from the exhaust gases of industrial activities to prevent its release into the atmosphere, then compress and convert it into a fluid that can be transported and either reused in specific operations such as enhanced oil recovery (EOR) or stored in depleted oil or gas reservoirs. Therefore, the captured CO_2_ must be safely transported to a storage facility or final utilization site in order to confine or use it without releasing it into the atmosphere. A key factor that could enable the rapid and large-scale implementation of CCTS is the repurposing of the existing underground pipeline infrastructure for CO_2_ transport. Transporting CO_2_ via pipelines has been recognized as the most efficient and economically viable method and has garnered significant attention from both industry and researchers. However, a key challenge identified in all analyses is ensuring adequate safety standards, not only during the capture and injection phases but particularly during transport across the territory. This involves studying the behaviour of the pipeline materials in contact with carbon dioxide in order to qualify them for such use. In particular, the composition of the transported mixture must be considered. Due to the nature of its source, the stream is not composed of pure carbon dioxide. The presence of other substances, such as non-condensable gases, residual unburned gases, and water vapour (which may condense), alters the mechanisms and severity of corrosion behaviour within the pipelines.

The aim of this review was to analyse the current state of knowledge on carbon capture, transport, and storage (CCTS), with particular emphasis on the compatibility of existing pipeline network materials with CO_2_ streams containing impurities, and how these may influence corrosion behaviour. The data considered in this review were collected through the systematic analysis of several databases, including Scopus, complemented by searches conducted using engines such as Google Scholar, with subsequent verification of the retrieved sources. The literature analysis was further supported by advanced tools for the graphical representation of recent research trends (e.g., Litmaps visualisations assisted by artificial intelligence).

### 1.2. CO_2_ Capture Techniques

**Figure 2:**
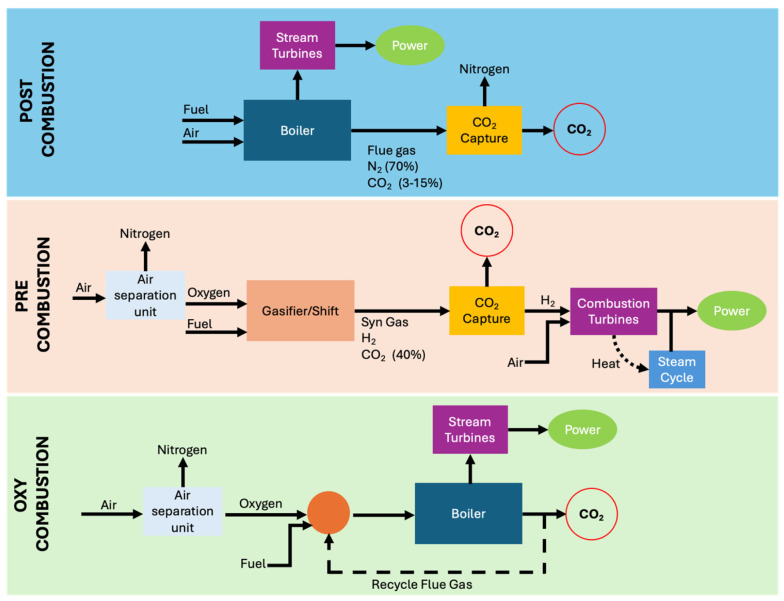
Scheme comparing the principal CO_2_ capture approaches, including post-combustion, pre-combustion, and oxy-fuel combustion technologies.

Currently, there are three main approaches for large-scale CO_2_ capture, as illustrated in Figure 2: pre-combustion, post-combustion, and oxy-fuel combustion. Each of these methods operates under different conditions, with distinct advantages and disadvantages, which are specifically outlined as follows.

- Pre-combustion: This method involves removing CO_2_ from fossil fuels upstream of the combustion process, resulting in a hydrogen-rich gas that can be used as a clean and versatile fuel in power generation plants or for alternative uses (transportation, basic chemicals, etc.). From an energy standpoint, the associated penalty is generally low, both because the process deals with limited flow rates and because it can operate under pressure, a condition that facilitates CO_2_ capture and reduces the energy cost of regenerating the sorbent agents [6,7].
- Post-combustion: This approach involves removing CO_2_ from flue gases after the combustion of the fuel source, e.g., extracting CO_2_ from the exhaust gases of power plants and industrial facilities. Post-combustion systems are the most technologically mature, thanks to the experience gained in the oil and gas sector and small-scale gas treatment applications. They are best suited for retrofitting existing plants, provided there is sufficient space available, given the large volumes involved. The main disadvantages are the high capital costs associated with the need to treat large gas volumes as well as significant energy penalties due to the regeneration phase [6,7].
- Oxy-fuel combustion: This third approach, among the most promising today, involves combusting the fuel source with nearly pure oxygen instead of air. This results in a flue gas stream rich in CO_2_ and free of nitrogen. After passing through pollutant removal units and a condensation section to remove water vapour, the stream can be sent to storage. This process already finds applications in the steel and glass industries and is currently being explored in power generation at the global level [6,7].

**Figure 3:**
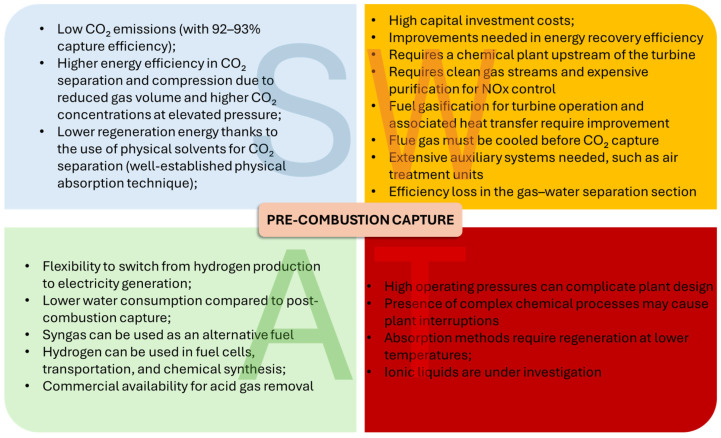
SWAT analysis for pre-combustion capture.

**Figure 4:**
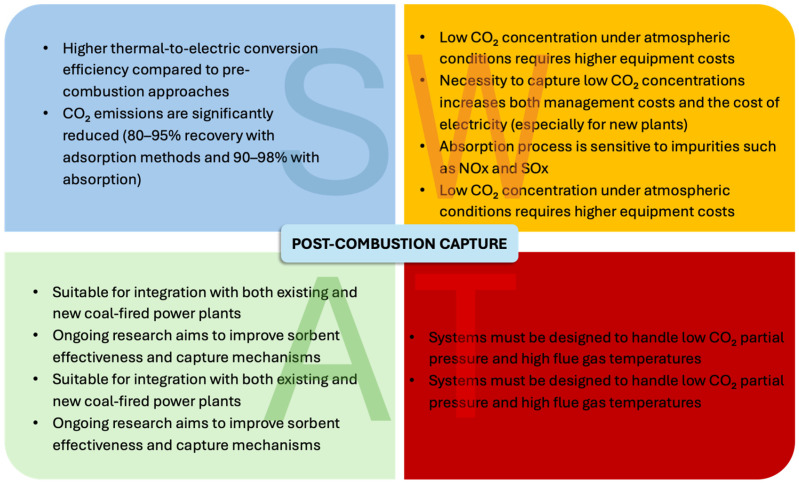
SWAT analysis for post-combustion capture.

The first two strategies discussed are historically the most widely adopted, largely due to the fewer limitations associated with retrofitting existing plants, as they do not require changes to the oxidant. The oxidant (usually air) generally does not represent a cost in these systems, and from an economic sustainability perspective, it is difficult to justify its replacement with alternative fluids. The SWOT analysis (Strengths, Weaknesses, Opportunities, and Threats) [6], presented in Figure 3 and Figure 4, compares pre- and post-combustion capture, highlighting the respective advantages and disadvantages of these two technologies.

## 2. Working Fluid Transported

### 2.1. Target Composition Criteria Within the Current Regulatory Framework

The widespread adoption of CCTS technologies has prompted standardization bodies to issue specific guidelines, such as those outlined in the ISO/FDIS 27913 [8]. These aim to regulate the operation and safety of such systems, with particular attention to plant management strategies, inspection scheduling, internal coating possibilities, and the composition of transported mixtures. All recommendations are intended to establish requirements that ensure safe operation of CO_2_ transport systems. The ISO/FDIS 27913 framework seeks to establish guidance through generalised, integrable, and verifiable principles rather than highly prescriptive rules. It builds upon accumulated industry experience derived from long-standing field applications, as reflected in earlier standards such as DNVGL-RP-F104 and the NORSOK standards, while simultaneously introducing broader and more theoretically grounded criteria. This ongoing evolution aims to strengthen consistency across practices and to promote harmonisation among standards within the oil and gas pipeline sector, thereby facilitating improved cooperation, interoperability, and integration across projects and regulatory frameworks. The standard highlights the well-known compatibility issues between the CO_2_ and the steel typically used in natural gas pipelines. As a result, it proposes target compositions, presented in Table 1 and Table 2, for gaseous and dense-phase CO_2_, respectively.

The focus is on components known to exacerbate corrosion mechanisms, such as the presence of H_2_S, SO_x_, NO_x_, non-condensable residues like CH_4_ and C_2_H_6_, and water. Beyond the compositional limits listed in the tables, the standard specifies the following: “The CO_2_ stream shall not contain impurities that may cause harm or damage to the pipeline, equipment, downstream systems, or storage reservoirs. Impurities not listed shall be brought to the attention of the pipeline operator for further assessment. CO_2_ streams from anthropogenic sources that do not meet specifications may be rejected at the discretion of the pipeline operator.”

Given the nature of the process and the fluid involved, it is therefore possible to encounter conditions in which the composition may be highly variable, depending on the type of upstream plant, the technology used for carbon dioxide capture, and the specific transport facility. In general, the fluids to be treated and transported may contain the following:

- Large volumes of O_2_, N_2_, H_2_, Ar, and H_2_O (in the form of vapour or condensate);
  ○ Particulates/dust that need to be mechanically separated (and which are generally absent during transport);
  ○ Traces of components that may act as catalyst poisons or be toxic to downstream process microorganisms, or even for human consumption. For example, acidic components such as H_2_S, SO_2_, SO_2_, HCl, HF, COS, CS_2_, CH_3_SH, HCN, NO, NO_2_/NO_3_, and Cl_2_; basic components such as NH_3_ and amines, whose presence is due to the flue gas purification process (amine-based processes being among the most common); combustible components such as CO, CH_4_, and organic substances; metallic components such as mercury, heavy metals (Ni, Cr, etc.), and alkali and alkaline earth metals (Na, K, Ca, Ba), which occur in the form of aerosols rather than particulates; volatile organic compounds such as aromatic hydrocarbons, olefins, aldehydes/organic acids, dioxins/furans, oils/greases, etc.

Moreover, the difficulty in maintaining unchanged composition over time and along the transport route as well as the variable temperature and pressure conditions influence metal–environment interfacial phenomena; the physical properties of pure carbon dioxide are altered by the presence of the aforementioned contaminants, resulting in a variation of the fluid’s properties, which may cause phase transitions at temperatures and pressures different from those of pure CO_2_, thus altering the characteristic corrosion mechanisms (generally worsening their severity). It is therefore appropriate to describe the transport fluid as a CO_2_ mixture.

The phase transition phenomena of the fluid are thus complex and difficult to predict. In particular, the standard suggests that “transient situations such as pipeline shutdown and cooling (especially in transport systems where a CO_2_ flow enters the pipeline at temperatures higher than ambient) must be considered. [Phase transition phenomena] may also occur during depressurization of a dense-phase transport system. Following such events, multiphase flow conditions are inevitable, and the operator must strive to minimize their duration.”

As described in the standard, the main aspects to be considered in the management of a pipeline under multiphase flow conditions are as follows:

- The maintenance of stable conditions; this may involve adjusting the injection capacity of the system (pipeline and well geometry, reservoir conditions, ambient temperature, compression and reservoir conditions, ambient temperature, compression and pumping equipment, etc.)
- The identification of flow conditions that can reduce hydraulic capacity (hydrate formation) and compromise system integrity (erosion, hydrate formation, corrosion potential, etc.)
- Maintaining temperature within an acceptable range; pressure reductions resulting from normal operations may lead to temperature drops due to evaporation of liquid CO_2_, affecting heat transfer and pipelines.

### 2.2. Phase Transitions and CO_2_ Phase Diagram

**Figure 5:**
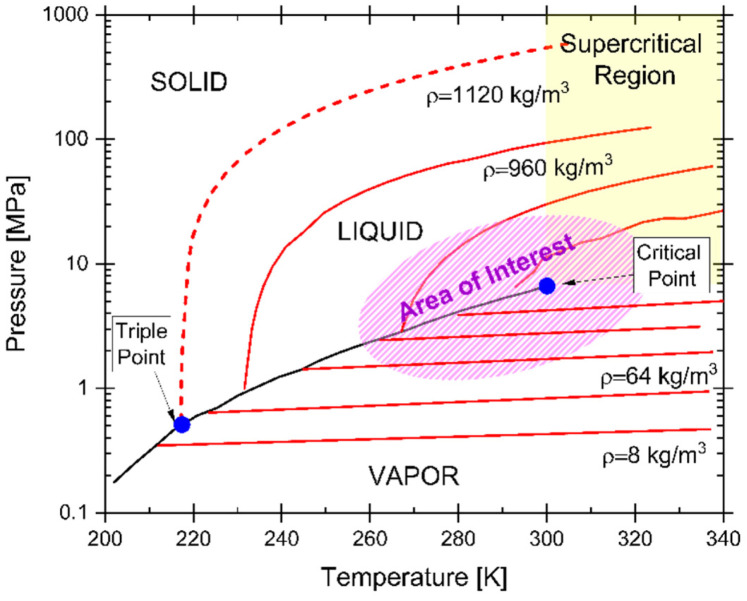
Phase diagram of pure CO_2_, illustrating phase boundaries between solid, liquid, and gaseous states as a function of temperature and pressure.

Even under controlled conditions, due to natural variations in temperature and pressure along the transport line and the variability of the incoming mixture, the fluid may undergo phase changes during pipeline transport. In particular, carbon dioxide can experience phase transitions along the pipeline, resulting in a biphasic state, e.g., a coexistence of liquid and gaseous phases. The morphology of the phase diagram is therefore an essential tool for predicting the behaviour of the transported fluid (and consequently its corrosion behaviour). The phase behaviour of pure carbon dioxide has been extensively studied and serves as a reference point. Figure 5 shows an example, including the vapour–liquid equilibrium curve, which extends from the triple point (216.59 K and 0.518 MPa) to the critical point (304.13 K and 7.377 MPa). A highly accurate equation of state for CO_2_ was proposed by Span and Wagner [9], valid over a wide range from 217 K to 1100 K and up to 800 MPa. Subsequently, several thermodynamic data sources have been published to further characterize CO_2_ behaviour under different conditions [10,11,12,13].

In the case study examined in this review, the pipelines considered are onshore buried pipelines, for which an average subsurface temperature of approximately 15–18 °C can be assumed, with internal pressures of around 40 bar. For offshore sections, the operating pressures are significantly higher (ranging from 100 to 300 bar), while the temperature remains relatively constant.

**Figure 6:**
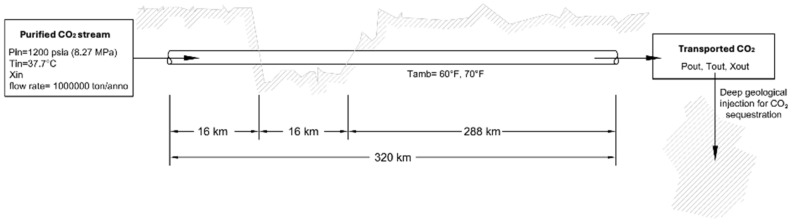
Schematic representation of the pipeline system analysis used in the model.

The literature provides several examples of operating parameters plotted on the phase diagram of pure CO_2_, which allow for optimal transport conditions [14], as shown in Figure 6. The graph presents the pressure and temperature conditions across the entire CO_2_ transport, storage, and utilization chain [3,13,15,16,17], operating in the so-called supercritical state (s-CO_2_). This specific condition ensures that the amount of CO_2_ transported per unit volume is maximized, as the fluid exhibits liquid-like density and gas-like viscosity [18]. For instance, the density and viscosity of s-CO_2_ at 50 °C and 10 MPa are approximately 395 kg/m^3^ and 0.03 cP, respectively [19].

For cost-effective CO_2_ transport, pipelines should ideally operate within a range of 20–50 °C and 5–15 MPa. However, ensuring full compatibility between these transport conditions and existing infrastructure can be challenging. In fact, the operating pressure range of conventional oil and gas pipelines is significantly lower than the optimal range for CO_2_ transport as reported in literature [17,20,21,22]. Nevertheless, altering the fluid’s operating conditions would reduce the economic viability of the transport process. Captured CO_2_ from combustion facilities is typically compressed above its critical point (7.4 MPa, 31 °C) to avoid biphasic flow regimes and thereby achieve optimal transport efficiency.

### 2.3. Effects of Impurities on the Physical Properties of the Fluid and on Facilities

The separation of impurities, if it is possible, represents both a cost and a complication in the CCTS process. For this reason, it would be reasonable to consider the direct injection of CO_2_ containing impurities into underground storage sites. Moreover, some emerging technologies aim at the co-capture and co-storage of multiple atmospheric pollutants alongside CO_2_, thereby reducing the extent of required treatment. However, the presence of impurities in the transported CO_2_ stream can significantly impact both pipeline network performance and corrosion behaviour within the infrastructure.

Impurities alter the physical properties of the fluid, consequently modifying the phase diagram and the critical transition values compared to those of pure CO_2_, as previously illustrated in Figure 6. Non-condensable impurities such as N_2_, O_2_, and Ar raise the saturation pressure of liquid CO_2_ and lower its critical temperature. As a result, a lower temperature and an additional overpressure are required to avoid undesirable biphasic flow during CO_2_ transport in pipelines. The type of pollutants present in the CO_2_ stream can therefore shift the operating pressure range, increasing the risk of entering biphasic regimes, as demonstrated by the phase envelopes of CO_2_ mixtures [23].

Indeed, studies in the literature have reported greater pressure drops during the pipeline transport of CO_2_ mixtures (e.g., impure CO_2_) compared to pure CO_2_ [18,24,25,26,27]. Naturally, the impurity levels depend on the capture and separation technology employed, as well as the CO_2_ source. CO_2_ derived from an Integrated Gasification Combined Cycle (IGCC) plant followed by a conventional amine scrubbing system tends to be contaminated mainly with carbon monoxide and hydrogen sulphide. In contrast, CO_2_ from oxy-fuel combustion processes is likely to exhibit elevated levels of argon, oxygen, and potentially nitrogen.

#### 2.3.1. Variation of the Critical Point: Canadian Case Study on Impurities

A relevant study on the transport of CO_2_ mixtures applied to real-case scenarios was conducted by Natural Resources Canada (NRCan), which has led several federal CCS programs. In addition to research and development projects focused on CO_2_ capture from coal-fired power plants and other industrial sources, NRCan has been involved in CO_2_ storage research initiatives, including injection, monitoring, measurement and verification, storage integrity assessment, and CO_2_ capacity estimation.

In this context, NRCan carried out a study supported by the International Energy Agency (IEA) and the U.S. Greenhouse Gas Center (US GHG), focusing on the effects of impurities in CO_2_ streams, with particular emphasis on saline formation storage technology, a strategy that appears to hold considerable promise [23].

The study analysed five different impurity mixtures combined with CO_2_ from various industrial sources and compared their behaviour to that of pure CO_2_, as summarized in Table 3.

In this study the behaviour of the various fluids was analysed using the Peng–Robinson equation of state, which is now widely adopted for estimating the phase envelope shifts of CO_2_ mixtures [31]. The results show that the critical temperature and pressure of the mixtures were different from those of pure CO_2_. The N_2_, O_2_, Ar, and H_2_ exhibit a pronounced effect, leading to an increase in the saturation pressure of the liquid phase and a reduction in the critical temperature. An extreme case is represented by mixture 5 (from Table 3), derived from combustion sources. In this scenario, the critical temperature decreases by approximately 10 °C, and the liquefaction pressure increases by more than 50 bar, compared to pure CO_2_. On the other hand, SO_2_ has the opposite effect: it leads to a reduction in saturation pressure and an increase in critical temperature, consistent with the high critical temperature of pure SO_2_ (157.6 °C). It is also worth noting that impurities present at low concentrations, such as CO and NO_x_, do not significantly influence the phase behaviour of CO_2_.

#### 2.3.2. Phase Diagram Variation: The Role of Water

Among all potential impurities, free water (H_2_O) is the most undesirable due to its capacity to induce hydrate formation and corrosion issues, particularly when associated with acidic gaseous components such as CO_x_, SO_x_, and H_2_S. When the water content exceeds its maximum solubility, it can form hydrates that potentially lead to pipeline blockages [3]. Moreover, even when water is the only impurity present, condensation due to temperature and pressure fluctuations along the pipeline can cause severe corrosion phenomena. It is well known that water presence is critical for corrosion processes in CCTS and EOR transport components, even under supercritical CO_2_ (s-CO_2_) conditions [32]. Consequently, it is essential to purify, dehydrate, and compress the gaseous CO_2_ prior to pipeline transport, as recommended by ISO/FDIS 27913, and to strictly control water concentrations in the transported fluid. Water is commonly present in all purified CO_2_ streams. After compression, the water content equilibrates to a saturation level determined by temperature and pressure. For example, if a CO_2_ stream is compressed from ambient pressure to 200 bar and then cooled to 35 °C for deep injection, the resulting mole fraction of water is approximately 0.0015 (1502 ppm) [25]. The water content can be reduced to acceptable levels through appropriate dehydration techniques prior to pipeline transport. The system composed by CO_2_ and water exhibits a broad region of fluid immiscibility and solid–fluid equilibria. The phase equilibrium conditions as functions of pressure (P), temperature (T), volume (V), and absolute humidity (X), defined as the mass of water vapour per volume of mixture, have been extensively studied and reported in numerous publications [33,34,35,36,37,38,39]. Recent thermodynamic property measurements for this system include the works of Novitskiy et al. [40] and Siqueira-Campos et al. [41]. The GERG-2008 equation of state (EoS) is the most widely used model for predicting the physical behaviour of the biphasic CO_2_–water system [42]. However, it is based solely on density data, which limits its accuracy in estimating phase equilibria particularly at the lower boundary to low relative humidity value. Furthermore, it has been estimated that if water concentrations at the compressor outlet exceed XCO_2_ ≈ 0.001, CO_2_ hydrate formation can occur in some sections of the surface infrastructure [33,38]. These hydrates may accumulate and, upon melting, locally increase the water concentration to levels where liquid water can collect in crevices, posing a significant corrosion hazard.

#### 2.3.3. Variation of Physical Properties of CO_2_ Mixtures Induced by Pipeline Transport: Pipeline Model

The undesirable phase transitions in a CO_2_ and pollutant mixture can be induced by the transport of the fluid itself along the pipeline. In fact, the inlet values of pressure, temperature, and relative humidity can vary due to pressure drops and the different positioning of the pipelines along the transport path. Therefore, the thermodynamic parameters that characterize the state of the fluid may change, causing state variations that can trigger phase transitions, which are often difficult to predict.

An interesting case study was analysed by Verma et al. [25], simulating the behaviour of a system with a purified carbon dioxide stream which, after compression and cooling in a post-cooler, must be transported and injected deep underground for CO_2_ abatement. The summarised scheme of the system simulated is shown in Figure 6.

The inlet values at the pipeline are considered known: pressure (Pin = 82.7 bar), temperature (Tin = 37.7 °C), vapour fraction, and mass flow rate (m = 1,000,000 tons/year). The room temperature is Tamb = 15.6 °C, and the case for Tamb = 21 °C is also analysed. Pressure drops due to friction in the pipeline are calculated using the Beggs and Brill correlations as well as the OLGA S. model.

For pure CO_2_, under supercritical inlet conditions and moderate ambient temperatures (15.6 °C), the fluid transitions to the liquid phase with relatively minor pressure drops and no vapour formation. The temperature of the CO_2_ stream decreases progressively along the pipeline until reaching ambient conditions. Under these ambient conditions, a pressure reduction is observed along the pipeline relative to the inlet. Phase change occurs only when the ambient temperature is increased, with the vapour fraction exceeding 40% at the outlet [25]. In contrast, for the CO_2_ + 4 mol% H_2_S mixture at a reduced inlet pressure (7.58 MPa), the phase behaviour alters significantly even at lower pressures compared to the pure CO_2_ case. Although the mixture initially enters the pipeline in a supercritical state, it transitions into the vapour–liquid region along the pipeline length. The vapour fraction increases progressively due to the combined effects of pressure reduction and the latent heat of vaporisation. This results in further cooling and increases frictional pressure losses, which in turn promote additional vapour formation through a positive feedback mechanism. Such behaviour is not observed in the pure CO_2_ case at comparable pressures, underscoring the critical influence of impurities on two-phase flow development and pipeline performance. Consequently, when the CO_2_ stream intersects the vapour–liquid boundary or enters the vapour region, frictional pressure losses become substantial and potentially prohibitive. The addition of small amounts of impurities such as H_2_S, N_2_, Ar, and O_2_ to CO_2_ does not significantly expand the pressure–temperature (P–T) immiscibility region [25]. Consequently, H_2_S does not cause notable variations in phase equilibrium effects, critical pressure changes, or compression requirements along the pipeline section.

This model was also applied to a CO_2_–water mixture. However, since the water content in the CO_2_ stream was limited to 0.0032 mol%, no impact of water vapour on the temperature or pressure profiles along the pipeline was observed. Therefore, based on the reviewed literature and under the proposed conditions, this value can be considered a threshold below which the thermodynamic parameters and the predicted fluid behaviour in the pipeline are comparable to those of pure CO_2_. Generally, the concentrations of impurities present in a CO_2_ mixture can vary significantly, primarily due to the diversity of the sources from which the streams to be transported originate. Compositional variation, especially under near-critical conditions, strongly influences pressure drop. Therefore, the models employed must consider the composition and the corresponding equation of state for operational design. The thermodynamic properties of pure CO_2_ are accurately described by the GERG-2008 equation of state (EoS). However, the behaviour of CO_2_ + H_2_S mixtures (and impurities in general) is difficult to estimate because data for this system at pressures above 20 MPa are scarce or unavailable. Consequently, GERG-2008 currently does not accurately reproduce fluid equilibria at low temperatures below 373 K, although sufficient data exist to potentially re-parameterize the model.

## 3. Phenomena of Corrosion Associated with CO_2_ Transport

CO_2_ streams transported at low to medium pressure in metallic pipelines can trigger corrosion phenomena known as sweet corrosion. These mechanisms have been studied for over 50 years, and there are now highly complex predictive models to estimate corrosion rates based on CO_2_ partial pressure, temperature [43,44,45], pH, dissolved salts, and the formation of more or less protective scales [46,47,48]. However, recent studies [49] have emphasized that these models are ineffective in predicting corrosion rates in the presence of high CO_2_ pressures and contaminants. Additionally, low-pressure and low-temperature operational conditions, as well as compositional changes associated with thermodynamic parameter variations along the transport, can cause condensates to accumulate and trigger sweet corrosion phenomena known as “top of the line corrosion” (TLC). Condensates of water vapour containing CO_2_ and other contaminants may form on the cooled inner walls of pipelines, thereby initiating aggressive corrosive processes [49,50,51].

### 3.1. The Role of Water in the Corrosion of Transported CO_2_ Mixtures

A key aspect in analysing sweet corrosion and TLC phenomena is the presence of water within transported fluids. The CO_2_ corrosion process generally follows three phases:

1. Dissolution of CO_2_
2. Hydration of CO_2_
3. Dissociation of carbonic acid

#### 3.1.1. Dry CO_2_ Flow (Absence of Water)

A primary distinguishing factor is whether water is present (in vapour–iquid or condensate biphasic conditions) within the transported CO_2_ flow, which alters the corrosion mechanisms. The influence of protective corrosion-product formation in an aqueous CO_2_ environment is highly complex, although the underlying chemical reactions in an H_2_O/CO_2_ system remain the same regardless of CO_2_ partial pressure [52,53].

In the literature, it has been widely demonstrated that dry CO_2_, that means with total absence of water, is not corrosive in an engineering sense. Numerous tests estimate corrosion rates under such conditions, for example the following:

- Short-term exposure tests [54,55]
- 24 h at 50 °C and 24 MPa on C1018 carbon steel [56,57]
- 96 h in supercritical dry CO_2_ at 80 °C and 12.5 MPa [57]
- 200 days at 9–12 MPa and 160–180 °C on AISI 1080 carbon steel yielded a corrosion rate of ~0.01 mm/year [58]
- After 12 years of service in high-pressure dry CO_2_ pipelines, carbon steel corrosion rates were 0.5–2.5 μm/year [59,60].

Flow-imposed experiments (e.g., 100 rpm at ~8 MPa and 35 °C) showed that corrosion rates for stainless steels 304 L and 316 L were 0.0008 and 0.0012 mm/year, while carbon steels X42 and X60 reached ~0.02 mm/year [61]. Therefore, carbon steels in a predominantly dry CO_2_ environment exhibit minimal corrosion. This conclusion is further supported by conductivity measurements (~3 × 10^−5^ S/m) of two orders of magnitude lower than those in water-saturated supercritical CO_2_ (~7 × 10^−3^ S/m) [62], indicating negligible corrosion in pure dry CO_2_. In summary, carbon steels exhibit negligible or no corrosion when exposed to dry CO_2_ environments.

#### 3.1.2. CO_2_ Flow in the Presence of Water

##### Mixtures of CO_2_ with Small Quantities of Water (Below Solubility Limit)

The behaviour of pipelines transporting CO_2_ with water present was analysed through USA field studies conducted from 1990 to 2001. These studies reported only two incidents attributable to corrosion phenomena [16], with generally low corrosion rates of ~0.00025–0.0025 mm/year [54,56]. Strict limits were imposed on allowable contaminants, particularly free water concentrations. For example, steel pipeline X60 exhibited corrosion rates < 0.001 mm/year at 14 MPa with 800–1000 ppm H_2_O [52]. In another study, a mixture of 600 g supercritical CO_2_ with 1.5 g H_2_O at 80 °C for 96 h yielded a corrosion rate of 0.0053 mm/year on X65 carbon steel [63].

Thus, if the water content remains below its solubility limit in CO_2_, no separate or water-rich aqueous phase forms. As a result, CO_2_ does not significantly dissolve in water, and carbonic acid (H_2_CO_3_), the primary driver of corrosion in ferrous materials, is not generated. In this condition the carbon steels do not experience significant corrosion under relevant pressure and temperature conditions [64,65,66,67,68].

Conversely, exceeding the solubility limit leads to rapid increases in corrosion rates due to liquid water phase presence in the CO_2_/H_2_O system. For example, mixtures with substantial water content (600 g supercritical CO_2_ and 100 g H_2_O [63]) at 50–130 °C for 96 h resulted in corrosion rates of 0.014–0.043 mm/year, nearly ten times higher than lower-water mixtures. Insufficient drying of liquid CO_2_ can lead to residual H_2_O accumulation, causing carbon steel corrosion and pipeline leaks [64]. Even with 100 ppm water, severe corrosion rates (up to 1.2 mm/year) were observed where residual water pools on carbon steel surfaces, creating aggressive local environments [64].

The corrosion rate data from various researchers [56,61,63,65,67,69,70,71,72,73,74,75,76] are summarised in Table 4, illustrating the extent of corrosion in steels across different water contents in supercritical or aqueous CO_2_ environments. The key conclusions drawn are as follows:

- Carbon steels and Cr-containing stainless steels show negligible or undetectable corrosion rates (<0.001 mm/year) in the absence of water
- Carbon steels incur mild corrosion (<0.1 mm/year) when water content in supercritical CO_2_ remains below saturation (undersaturated conditions)
- Carbon steels suffer corrosion rates exceeding 0.1 mm/year, (and even reaching 1 mm/year) when in water-saturated supercritical CO_2_.

##### Mixtures of CO_2_ with Saturated Aqueous Phase

As the water content in the CO_2_/H_2_O mixture increases, the system gradually transitions from a CO_2_-rich phase to a H_2_O-rich phase. This process can be supported by the mutual dissolution of supercritical CO_2_ (s-CO_2_) into H_2_O and vice versa. When the water content in CO_2_ is high, the primary phase of the system consists of free water. Supercritical CO_2_ conditions have been widely studied in the literature, and their behaviour can be extrapolated to cases of CO_2_ transport under non-supercritical conditions.

To compare the difference in corrosion rates of carbon steels in s-CO_2_ environments between CO_2_-rich and H_2_O-rich phases, the corrosion behaviour of carbon steel exposed for 24 h at 50 °C under pressures ranging from 4.6 to 8 MPa was analysed in environments with either predominant s-CO_2_ or predominant H_2_O content [76]. It was found that in both environments, the corrosion rate does not significantly depend on CO_2_ pressure, but rather on the presence of water. Specifically, in a CO_2_-rich environment, the corrosion rate is approximately 0.4 mm/year, whereas in an H_2_O-rich environment, the rate reaches about 20 mm/year. The corrosion rates of carbon steels under these conditions have been reported by various researchers, as reported in Table 5 [63,64,65,75,76,79,80]. It can be observed that the corrosion rate in H_2_O-rich systems was nearly two orders of magnitude higher than in CO_2_-rich systems.

However, literature sources [70,81] have also shown that the corrosion rate increased when the water content was raised from 500 to 1000 ppmv in flow loop tests. Interestingly, it was also reported that further increasing the water content from 1000 to 2000 ppmv resulted in a decrease in the corrosion rate.

Under s-CO_2_ conditions, the N.O.R.S.O.K. [79] and K.S.C. [83] models (commonly used to predict carbon steel corrosion rates in CO_2_ environments) are no longer applicable. For example, in studies involving X65 steels [80], the N.O.R.S.O.K. and K.S.C. models estimated corrosion rates of 10 to 17 mm/year, whereas experimental tests reported rates between 1 and 6 mm/year. The values predicted by the two models are significantly higher than those measured and are considered overly conservative.

### 3.2. Corrosion Mechanism: Top of the Line Corrosion (TLC)

Top of the line corrosion (TLC) operates through a complex mechanism that results in severe localized corrosion, frequently affecting critical components. In CO_2_-dominated environments, the types of localized attack are commonly the following:

- pitting corrosion, when attack initiates at small defects in the passive corrosion-product layer where the base metal interfaces with the environment
- mesa corrosion, when steel is not corroded uniformly but presents surface pits often wide and flat-bottomed, surrounded by corrosion products [84].

Field applications show that carbon-steel pipelines used for CO_2_ transport typically have inner diameters ranging from 14 to 22 inches. The lower portion (approximately one-third of the external surface) is buried in soil or seabed and is often thermally insulated, whereas the upper part may not be buried and thus inadequately insulated.

A typically hotter fluid flows inside the pipeline, while along the uninsulated top, heat exchange results in condensate formation. Internal temperatures may reach up to 90 °C under certain conditions [46], making internal–external heat exchange significant. Consequently, high wall condensation rates can occur locally. The corrosion mechanism is governed by the local equilibrium between condensation droplets and the pipe metal. Condensation droplets may become saturated with FeCO_3_ (siderite). At high temperatures and low condensation rates, such saturation allows for the formation of a dense FeCO_3_ deposit that substantially reduces the corrosion rate [49,50]. However, the effectiveness of siderite as a protective layer depends on droplet renewal frequency and the overall aggressiveness of the environment. These phenomena are particularly relevant at dew point “cold spots”, characterised by a high water condensation rate (WCR), where pipeline thermal insulation is compromised.

#### 3.2.1. Factors Influencing TLC Mechanisms

TLC corrosion phenomena [49] arise under several conditions:

- Pipeline walls separating a hot internal environment from a colder external one (e.g., seawater or soil)
- High internal condensation rates
- High internal temperatures
- High CO_2_ partial pressures
- High water-vapour loads.

As previously discussed, the liquid–vapour equilibria of transported mixtures depend on CO_2_ composition and contaminant concentrations, potentially inducing unintended phase transitions at temperatures and pressures not usually associated with such phenomena. However, some key parameters that characterise the corrosion rate include gas temperature, CO_2_ partial pressure, and condensation rate.

##### Gas Temperature

Gas temperature influences the condensation rate and corrosion-product nature, thus affecting corrosion speed. At low gas temperatures, the condensation and corrosion rate are limited. At higher temperatures, corrosion products may precipitate to form protective deposits that mitigate corrosion. Very low gas temperatures can reach critical condensation rates, but FeCO_3_ formation kinetics are unfavourable, leading to supersaturation without protective precipitates and localized attacks that eventually arrest [84]. Around 40 °C, FeCO_3_ solubility increases, leading to porous, non-protective siderite layers [49]. Corrosion rates then rise in the initial days and then plateau. If the gas temperature is insufficient to form a coherent protective layer, corrosion occurs uniformly [84]. At high temperatures (70 °C), initial corrosion rates are high due to fast condensation, but decline over time as dense carbonate layers form, although these conditions are not typical of transport scenarios.

##### CO_2_ Partial Pressure

CO_2_ partial pressure directly affects corrosion-product scale formation. Low CO_2_ partial pressure (0.13 bar) does not favour protective FeCO_3_ scabs, resulting in moderate, steady corrosion. In contrast, high partial pressures facilitate protective siderite formation: initial corrosion rates are high, declining over time as exposure increases and FeCO_3_ becomes supersaturated. High CO_2_ pressure impact is most marked at high condensation rates, where the condensate is hard to saturate and the pH is more affected by CO_2_ pressure. Elevated CO_2_ partial pressure increases corrosion by lowering the pH through enhanced hydration and dissociation of CO_2_:

$$CO_{2}+H_{2}O\to H_{2}CO_{3}\to H^{+}+HCO_{3}^{-}$$

**Figure 7:**
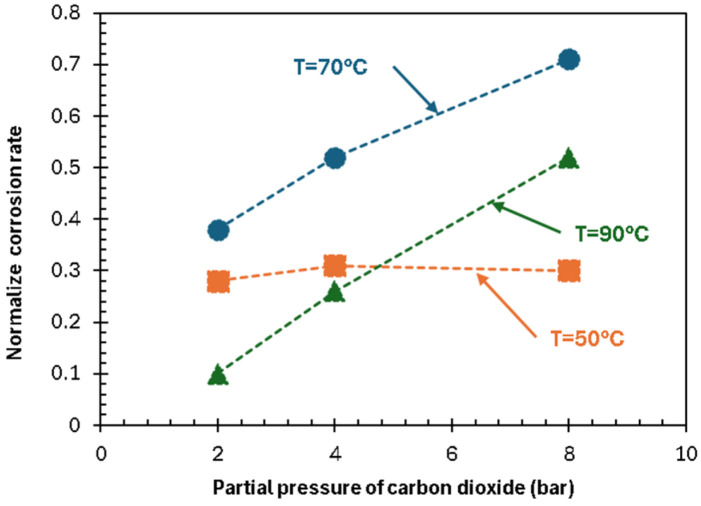
Effect of CO_2_ partial pressure (pCO_2_) on the corrosion rate of carbon steel under top of the line corrosion (TLC) conditions, adapted from [50].

Vitse [46] studied CO_2_ pressure effects (1–8 bar) at 50, 70, and 90 °C and varied sub-cooling (difference between fluid and wall temperature). The effect of CO_2_ partial pressure is pronounced at high condensation rates (70–90 °C with high sub-cooling), much less so at 50 °C or without sub-cooling (Figure 7). Other studies [85] similarly show that the corrosion rate is visibly influenced by the CO_2_ partial pressure when paired with high condensation rate. At low condensation, corrosion-product saturation inhibits ion transfer to/from metal, reducing the CO_2_ pressure effect on the corrosion rate.

##### Water Condensation Rate

The condensation mechanism occurring within the pipeline walls due to the transport of hot fluid can be described by a characteristic condensation curve that includes a dropwise region, a film wise region, and a transition region [86].

At constant vapour velocity, dropwise condensation occurs at very low surface sub-cooling; this mode may persist even under high sub-cooling and large heat flux conditions. However, when sub-cooling becomes sufficiently large, the condensation rate exceeds the droplet detachment rate, leading to the accumulation of liquid on the surface and the formation of a continuous condensate film (filmwise condensation). Dropwise condensation is a complex phenomenon involving a series of subprocesses that constitute the dynamic “life cycle” of the droplet itself.

Key factors influencing condensation rate under TLC include the following:

- Gas temperature
- Surface sub-cooling
- Incondensable gas concentration
- Gas velocity
- System pressure
- Pipeline inner diameter.

**Figure 8:**
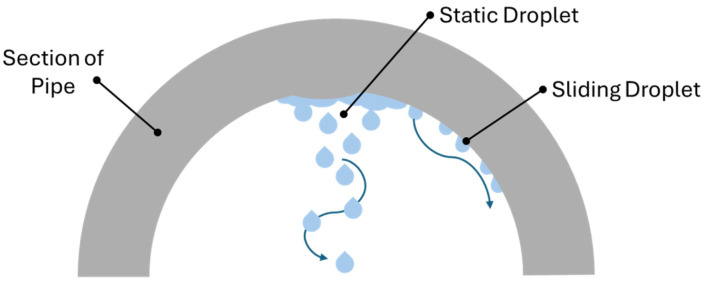
Schematic representation of dropwise condensation modes on the upper walls of a pipeline, illustrating static droplets and sliding droplets along the internal surface of the pipe.

The gas velocity and pipe diameter significantly influence surface wetting kinetics (Figure 8). At low gas velocity or large diameters, droplets may grow large and fall, creating localised acidic droplets that promote localized attack. At higher gas velocity, condensate rivulets slide along the wall forming film-like morphology, leading to more uniform corrosion.

To initiate TLC, a critical condensation rate (CCR) between 0.15 and 0.25 mL m^−2^ s^−1^ must be reached in real transport applications [47]. This range serves as an engineering threshold for moist CO_2_ transport, though it may not hold for all real conditions. Generally, condensation rates well above CCR trigger severe corrosion; the pH becomes more sensitive to CO_2_ partial pressure since Fe ions (normally buffering pH) cannot compensate. Conversely, condensation rates below CCR favour FeCO_3_ saturation and protective film formation, raising the pH and slowing the CO_2_ corrosion kinetics. At rates just above CCR, the corrosion rate decreases relative to the critical rate due to film formation [50].

Additionally, gas velocity alters the condensation rate: Vitse and Nešić [46] found that at both high and medium temperatures (50–90 °C), the condensation rate significantly decreases as the gas velocity declines (from 8 to 2 m/s); the mass transfer from the gas phase is less efficient at low flow turbulence.

#### 3.2.2. TLC Mechanisms in CO_2_-Dominant Environments

Initially, condensate droplets on steel surface produce high corrosion rates that stabilise as protective carbonate layers form—similar to sweet corrosion. However, TLC exhibits the following distinctive features:

- FeCO_3_ saturation forms a highly protective scale during early stages, limiting Fe ion release and metal dissolution. Yet continuous renewal of condensate (lacking Fe ions) sustains corrosion.
- Equilibrium is reached only when FeCO_3_ saturation approaches unity; the Fe ion input from steel matches the dilution by fresh condensate.
- High condensation rates promote rapid solution renewal at walls, hindering stable protective film development and maintaining high corrosion rates; conversely low condensate mass flux supports protective scale formation, yielding low but non-zero corrosion rates.
- Temperature strongly affects FeCO_3_ deposition kinetics. At low temperatures (40 °C), the corrosion rate is steady but moderate due to inhibited protective film formation. At temperatures > 70 °C, protective film quickly grows but forms surface cracks. Defects in the coating foster localized TLC attacks, which are eventually limited over time: the electrolyte within fissures promotes film growth, partially occluding defects. Localized attack rates can reach 5–10 mm/year depending on the condensation rate and environmental aggressiveness.

### 3.3. Role of Other Impurities on Corrosion Behaviour

As previously discussed, the CO_2_ transported in the context of CCTS, due to the various emission sources and the limited capacity for stream purification, may contain impurities such as O_2_, SO_x_, and NO_x_. These alter the thermodynamic properties of the fluid and thus promote corrosion of steels not only at low partial pressures but also under supercritical conditions. Some considerations can be made regarding specific impurities by analysing their individual roles in the associated corrosion mechanisms. However, it is important to note that complex mixtures composed of multiple impurities may affect corrosion phenomena differently compared to the effects observed with single species.

#### 3.3.1. Oxygen (O_2_)

The effect of O_2_ in CO_2_ on steel corrosion rates, particularly under s-CO_2_ conditions, has been widely investigated in the literature [61,64,65,70,74,87,88,89], as summarized in Table 6. In general, the corrosion rates of carbon steels in water-saturated CO_2_ environments containing O_2_ exceed 0.1 mm/year.

Small amounts of oxygen (100 ppm) in experiments conducted at 7.58 MPa and 40 °C with 1000 ppm of water in undersaturated s-CO_2_ showed no significant effect on the corrosion rate of carbon steels [74]. Similar results were obtained even at higher O_2_ concentrations, as long as the water content remained below its solubility limit in s-CO_2_ under the test conditions [64,70]. However, in water-saturated s-CO_2_ environments, the addition of O_2_ can lead to severe corrosion.

Studies on the effect of different O_2_ contents (0%, 2%, 4%, and 6%) on the corrosion of X65 steel in water-saturated CO_2_ at 8 MPa and 50 °C after 24 h of exposure showed an increase in corrosion rate, reaching a maximum value of 1 mm/year with 4% O_2_ addition [65,76].

In the absence of O_2_, the steel surface is covered with a dense, protective siderite (FeCO_3_) layer. When O_2_ is present in the system, it can inhibit FeCO_3_ formation and promote the formation of porous iron oxides with reduced protective capability, resulting in increased corrosion rates. Consequently, in water-saturated CO_2_ environments containing O_2_, carbon steels (e.g., X42 and X60) become more susceptible to corrosion [61].

When varying the temperature in dense-phase CO_2_ environments containing 50 vol% water at 10 MPa with different O_2_ concentrations (0, 100, and 200 ppm), it was observed that the addition of O_2_ increases the corrosion rate of carbon steel X65 by 50–120% [66]. At 50 °C, Fe^2+^ reacts with O_2_, forming iron oxides, which results in a lower Fe^2+^ concentration in the dense CO_2_ phase compared to that without O_2_. The reduced Fe^2+^ content destabilizes the protective siderite layer and leads to local failure of the FeCO_3_ film. As a result, severe localized corrosion can occur with rates up to 17 mm/year.

An important aspect to consider is the exposure time, which significantly affects the evaluation of oxygen’s influence on corrosion rates. For example, in tests with carbon steels exposed to water-saturated s-CO_2_ at 50 °C and 8 MPa, it was found that during the initial corrosion period the addition of 4% O_2_ had little to no effect, with corrosion rates of 19.2 mm/year without O_2_ and 19.3 mm/year with 4% O_2_. However, when the immersion time was extended to 120 h, the corrosion rate with 4% O_2_ (14.1 mm/year) was notably higher than that without O_2_ (10.6 mm/year) [65,76].

#### 3.3.2. Nitrogen Dioxide (NO_2_) and Sulphur Dioxide (SO_2_)

The effects of equal concentrations of O_2_, SO_2_, and NO_2_ on the corrosion of carbon steel in the s-CO_2_/H_2_O system at 40 °C and 7.58 MPa have been compared [69,70,74,77]. It was observed that 100 ppm of O_2_ had no effect on the corrosion rate of carbon steel, whereas the corrosion rate increased from 2.3 to 4.6 mm/year in the presence of 100 ppm of SO_2_. However, with 100 ppm of NO_2_, the maximum corrosion rate of carbon steel increased significantly, reaching up to 11.6 mm/year.

The effect of NO_2_ can lead to significantly higher corrosion rates of carbon steel (0.06–1.6 mm/year [76]) compared to the same concentration of SO_2_ (≤0.02 mm/year). In particular, the corrosion rate decreases with decreasing NO_2_ content but remains higher in the s-CO_2_/NO_2_/H_2_O environment. This suggests that NO_2_ dissolves in water to form HNO_3_, leading to a sharp decrease in pH. HNO_3_ exhibits a strong oxidizing effect on Fe^2+^ ions, resulting in the formation of a powdery, rust-like corrosion product. This corrosion film is soft and less protective, thus significantly increasing the corrosion rate of carbon steel in the presence of NO_2_ [67]. Therefore, in a CO_2_ system containing water, the following apply:

- O_2_, SO_2_, and NO_2_ can accelerate the corrosion of carbon steels in CO_2_/H_2_O environments. Among the three impurities at the same concentration, NO_2_ has the most significant effect on the corrosion rate of carbon steel, followed by SO_2_ and O_2_
- For SO_2_, corrosion can occur in the CO_2_/SO_2_/H_2_O system even when the water content is well below the solubility limit of CO_2_ in water. The corrosion rate increases with the concentration of SO_2_.

#### 3.3.3. Effects of Real Impurity Mixtures

##### Effect of Mixed Impurities

The corrosion behaviour associated with mixtures of multiple impurities under various pressure and temperature conditions may differ significantly from that estimated in previous cases with single-species environments. This deviation arises from interactions among the different species in the environment. Several research groups worldwide have published systematic studies with extensive datasets on corrosion, including Choi et al. [63,75,76,89], Xiang et al. [91,92], Hua et al. [93,94,95], Xu et al. [96,97], Sun et al. [98,99,100], Yevtushenko et al. [101], and Wei et al. [32,102].

**Figure 9:**
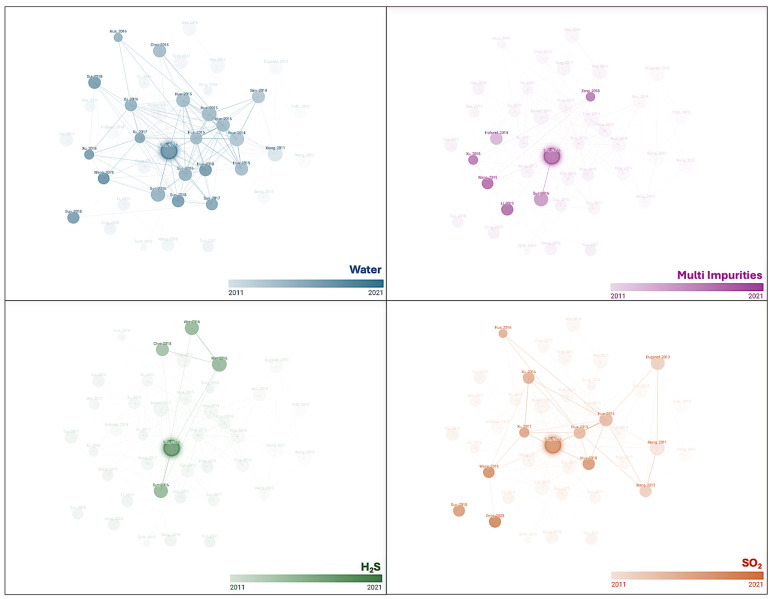
Literature data illustrating the most commonly studied environments for steels in supercritical CO_2_, including the presence of water, H_2_S, SO_2_, and impurity mixtures. The dataset is based on a comprehensive review of the most recent literature, supported by AI-assisted tools to enhance data collection and analysis.

For instance, Figure 9 presents a map of corrosion research data in s-CO_2_ environments, highlighting the most studied environments with the presence of water and different impurities. The effect of water on the corrosion rate has been extensively studied, as previously discussed, while the influence of SO_2_ and H_2_S has also been investigated. There remains a need to examine the combined effect of multiple impurities on corrosion behaviour. In recent years, a renewed interest has emerged in understanding how complex impurity interactions influence corrosion processes, reflecting the increasingly realistic conditions found in industrial and environmental settings. Despite these extensive efforts, no solid conclusions have yet been reached due to conflicting results reported by different groups. Factors such as differing exposure durations and cleaning methods have influenced the estimation of the corrosion rates.

Some studies have attempted to simulate realistic CO_2_ transport conditions by adding small amounts of impurities to the main CO_2_ stream and testing their effects on pipeline steels. For example, considering a flow of 4 L/h of s-CO_2_ containing 1000 ppmv H_2_O (decreasing to 500 ppmv, undersaturated) at 10 MPa and 60 °C, with additional O_2_ (8100 ppmv), SO_2_ (70 ppmv), NO_2_ (100 ppmv), and CO (750 ppmv) [103], it was observed that after 168 h of exposure the generalized corrosion rate for carbon steel (X52) was approximately 0.00003 mm/year. In contrast, pitting corrosion was observed for a CrMo alloy (UNS G41400) [103,104], which could be mitigated by appropriate heat treatment of the material [105].

For carbon steels X52 (L360NB) and X70 (L485MB) under s-CO_2_ conditions (11 MPa and 60 °C), the addition of O_2_ increased the corrosion rate [106]. The corrosion rate for X70 decreased significantly with a reduction in O_2_ content, while for X52, O_2_ concentration had no noticeable effect. Nonetheless, the corrosion rates for both carbon steels remained below 0.1 mm/year, indicating that a small amount of water in s-CO_2_ (undersaturation) has limited impact on corrosion. Additionally, adding a small amount of CO (50 ppmv) or SO_2_ (70 ppmv) to the mixture did not change the corrosion behaviour. However, the addition of 100 ppmv NO initiated corrosive phenomena [106]. The presence of multiple impurities significantly increases the average corrosion rate, up to twice as high in some cases. The complex synergistic interactions among CO_2_, O_2_, H_2_S, and SO_2_ contribute to the highest observed corrosion rates [107,108].

##### Amine Treatment and Effects on Corrosion Rates

The most widely adopted method for CO_2_ separation from flue gases is the amine-based process [109], favoured due to the relatively low cost of ammonia used as a sorbent and its high CO_2_ loading capacity. This has prompted the development of numerous studies aimed at improving its industrial application [110]. However, it is possible for the CO_2_ stream downstream of treatment to be contaminated with residual amine or ammonia.

Studies have investigated the effects of amine residues (100 ppm water + 100 and 1000 ppm of amine) on the water solubility limit in s-CO_2_, the pH value of the condensed aqueous phase, and the corrosion rate of carbon steels [77]. Amines significantly increase the pH of the condensed water, thereby reducing the corrosion rate of carbon steel: for 100 ppm of amine, corrosion rates were estimated at 0.1 mm/year, and for 1000 ppm at 0.01 mm/year.

Moreover, the addition of 50 wt% ethylene glycol (MEG) in a NaCl solution saturated with CO_2_ at 8 MPa and 50 °C decreased the corrosion rate of X65 steel from 4.6 to 1.7 mm/year [32].

## 4. Conclusions

It is important to consider the supply chain and origin of flue gases as their composition is strongly dependent on several operational factors, including flame temperature, fuel type, and process conditions. In addition, flow rates and the stability of both mass flow and gas composition may vary significantly, further influencing downstream behaviour.

It can be concluded that, in the absence of water, CO_2_ (whether in supercritical, gaseous, or liquid form) does not generally induce significant corrosion under low-temperature conditions or result in negligible corrosion rates. However, in the presence of water, the CO_2_-induced corrosion mechanism becomes significantly more complex and depends on several interacting factors. These include the partial pressure of CO_2_, which in the absence of other species governs the pH of the aqueous phase, as well as the pH itself, which can be modified by the presence of other chemical species affecting equilibrium conditions through changes in CO_2_ solubility. Temperature also plays a key role, as it influences both the solubility of CO_2_ in the aqueous phase and the nature, morphology, and stability of the corrosion-product scales. Furthermore, the presence of additional species can alter cathodic reaction kinetics, modify pH, and influence the composition and protectiveness of the corrosion scales. Flow rate is another critical parameter, as it affects both the formation and the mechanical stability of the corrosion layers.

Under these conditions, several predictive models for the corrosion rate of carbon steel are available. In addition, more advanced approaches explicitly consider corrosion-product formation and its coupling with microstructural evolution. However, the development of reliable predictive models remains challenging due to the complexity associated with CO_2_ phase behaviour and phase transitions under transport conditions.

In wet gas environments, water condensation is a key factor governing corrosion severity. In CO_2_–water systems, the role of impurities becomes particularly significant. For instance, small amounts of oxygen (approximately 100 ppm) under experimental conditions of 7.58 MPa and 40 °C with 1000 ppm water in supercritical CO_2_ (undersaturated conditions) have been shown to have no substantial effect on the corrosion rate of carbon steels. In contrast, O_2_, SO_2_, and NO_2_ can accelerate corrosion in CO_2_/H_2_O systems, with NO_2_ exhibiting the strongest effect, followed by SO_2_ and O_2_ when compared at similar concentrations. For SO_2_, in particular, corrosion can occur even when water content is below the solubility limit of CO_2_ in water, and the corrosion rate increases with increasing SO_2_ concentration.

The presence of impurities also alters the gas–liquid equilibrium boundaries compared to pure CO_2_ systems. Acidic impurities generally enhance corrosion in s-CO_2_ environments when water is present, with the exception of H_2_SO_4_. The addition of HNO_3_ may further promote localized corrosion phenomena such as pitting. On the other hand, alkaline species such as amines and NaOH tend to reduce the corrosion rate of carbon steels. Amines, in particular, significantly increase the pH of condensed water, leading to reduced corrosion rates, with reported values decreasing from approximately 0.1 mm/year at 100 ppm amine to as low as 0.01 mm/year at 1000 ppm. Additionally saline impurities such as Cl^−^, NO_3_^−^, and SO_4_^2−^ can significantly increase corrosion rates in s-CO_2_ environments and may also promote localized corrosion mechanisms.

Future research should focus on validating existing predictive models through dedicated in-field or industrial-scale experiments, as laboratory data alone remain insufficient to fully capture real operating conditions. In particular, the development of reliable predictive approaches is hindered by the complexity of CO_2_ phase behaviour and phase transitions under pipeline conditions. A more detailed understanding of how phase diagrams are modified in the presence of impurities would be highly beneficial, as this could allow the identification of key, directly measurable parameters that can serve as practical indicators for corrosion risk assessment in real systems.

## Figures and Tables

**Table 1:** Composition requirements for the CO_2_ gas phase as specified by ISO/FDIS 27913, shown as a radial schematic representation of the allowable impurity distribution in the system.

Component	Hazard(s) in a CCU Context	Units	Limit
CO_2_	Asphyxiation, and can act as a toxicant at high concentrations	mol%	>95.0
N_2_ ^a^	Enhances the potential for ductile fracture Occupies store pore space inefficiently	mol%	≤4.0
H_2_ ^a,b,c^	Enhances the potential for ductile fracture and hydrogen induced crack propagation Affects the size of the multi-phase zone	mol%	≤1.0
Ar ^a^	Occupies store pore space inefficiently, enhanced potential for running ductile fractures	mol%	≤4.0
CO ^a^	Health and safety: toxic gas	mol%	≤0.2
Methane ^a^	Occupies store pore space inefficiently	mol%	≤4.0
Ethane ^a^	Occupies store pore space inefficiently	mol%	≤4.0
Propane and other aliphatic hydrocarbons ^d^	Liquid drop-out is possible	mol%	≤0.15 in total
H_2_O	Enables corrosion of carbon steel	ppm mol	≤50
O_2_ ^b,e^	Enables oxidation of carbon steel Enhances bacterial growth in storage strata Other chemical reactions (e.g., with NO_x_, SO_x_, H_2_S)	ppm mol	≤10
NO_x_ (NO, NO_2_) ^f^	Degradation of store caprock Takes place in the production of nitric and sulfuric acid	ppm mol	≤10
SO_x_ (SO, SO_2_, SO_3_) ^g^	Degradation of store caprock Reactions with NO_2_ can produce sulfuric acid	ppm mol	≤10
H_2_S ^h^	Health and safety: toxic gas with foul odour	ppm mol	≤5
COS	Health and safety: toxic gas with foul odour	ppm mol	≤100
CS_2_	Health and safety: toxic gas with foul odour	ppm mol	≤20
NH_3_	Can react to form solid ammonium carbamate and other ammonium salts	ppm mol	≤10
BTEX ^i^	Health and safety: toxic	ppm mol	≤15 in total
Methanol	Can introduce a liquid corrosive phase	ppm mol	≤350
Solid particulates ^j,k^	Can reduce store permeability. Damage to compressor components	mg/Nm^3^	≤1 in total
Toxic metal ^j^	Health and safety: toxic	mg/Nm^3^	≤0,15
VOCs ^l^	Health and safety: toxic	mg/Nm^3^	≤48 in total
Acid forming compounds ^m^	Enables corrosion of carbon steel	mg/Nm^3^	≤150 in total
Amines ^n,o^	Can introduce a liquid corrosive phase	ppb mol	≤100 in total
Glycols ^p^	Enables aqueous corrosion of carbon steel		—
Nitrosamines and nitramines ^q^	Health and safety: bio-toxic	μg/Nm^3^	≤3 in total
Naphthalene	Health and safety: toxic	ppb mol	≤100
Dioxins and furans ^r^	Health and safety: toxic	ng/Nm^3^	≤0.02 in total

^a^→Combined total ≤ 5.0 mol%. ^b^→Risk of acid drop-out with hydrogen > 100 ppm mol, if levels of SO_2_, H_2_S, O_2_, and NO_2_ are much higher. ^c^→Avoidance of SCC. ^d^→Heavy hydrocarbons (C3+) shall not shift the dew point below that of pure CO_2_. ^e^→Presence of O_2_ influences the formation of strong acids and elemental sulphur, and increases the sensitivity to sulphur-induced stress corrosion cracking. ^f^→Separating out the different components of NO_x_ can allow higher levels of some species. ^g^→Separating out the different components of SO_x_ can allow higher levels of some species. ^h^→H_2_S tends to form SO_2_ and can form elemental sulphur, reacting with O_2_ if present in sufficient levels. ^i^→Separating out the different components of BTEX can allow higher levels of some species. ^j^→The maximum size of the particulate is 1 µm. ^k^→To include: Ash, dust, Na, K, Mg, Cr, Ni, Cd, Hg, Tl, Pb, As, and Se. ^l^→To include: formaldehyde, acetaldehyde, dimethyl sulphide, ethanol. ^m^→To include: Cl_2_, HF, HCl, HCN. ^n^→The maximum size of the liquid droplet is 2 µm. ^o^→To include: MEA, MDEA, DEA, AMP, piperazine, and any proprietary mixture containing any amine. ^p^→To include: TEG, MEG, DEG, propylene glycol, dimethyl ethers of polyethylene glycol. ^q^→To include: NDMA, NMEA, NDEA, NDELA, NPIP, NMor. ^r^→To include: PCDD, PCDF.

**Table 2:** Composition requirements for CO_2_ in the dense phase as specified by ISO/FDIS 27913 including the allowable limits for impurities.

Component	Notes	Units	Limit
CO_2_	Dry basis	mol%	>95.0
N_2_	Total non-condensables to be <5 mol%	mol%	a,b
H_2_		mol%	≤1
Ar		mol%	a,b
CO		mol%	≤0.7
Methane		mol%	
Ethane		mol%	
Propane and other aliphatic hydrocarbons	Total hydrocarbons to be <5 mol% and a dew point of product with respect to hydrocarbons to be <−20 °C.	mol%	≤1
H_2_O	The limit for water may be higher (e.g., 630 ppm mol) if the CO_2_ stream contains very low levels of O_2_, NO_x_, and SO_x_ (e.g., geological CO_2_).^b^	ppm mol	≤100
O_2_		ppm mol	≤10
NO_x_ (NO, NO_2_)		ppm mol	≤1.5
SO_x_ (SO, SO_2_, SO_3_)		ppm mol	≤1
H_2_S		ppm mol	≤55
Total sulphur		ppm mol	≤50
Solid particulates		ppm wt	≤1
Mercury		ng/L	≤5
Amines		ppm wt	≤1
Glycols	Must not be present in a liquid state at the temperature and pressure conditions of the pipeline.	ppm mol	≤50
Compressor lube oil carry-over		ppm wt	≤50 ppmw
Liquids	CO_2_ stream shall be free of liquids at delivery conditions and shall not produce condensed liquids in the pipeline at pipeline temperature and pressure.		

a→Impurities causing harm of damage to pipelines, equipment, downstream systems, or reservoirs. b→It is possible, with a water content of 100 ppm mol, for water drop-out to take place during depressurization (e.g., for maintenance). If this operation is planned, then a gas phase specification should be considered to avoid aqueous phase formation.

**Table 3:** List of CO_2_ mixtures studied by Natural Resources Canada (NRCan) from different plants.

No.	Source and Type of Plant	Transported Mixture	Reference
1	CO_2_ stream from oxy-fuel combustion in a fluidized bed combustor at the CanmetENERGY pilot plant	5.2 vol% O_2_, 221 ppm CO, 1431 ppm SO_2_, 243 ppm NO	[28]
2	CO_2_ stream from a zero-emission process proposed by CanmetENERGY	1.05% CO, 1.7% SO_2_, 0.32% H_2_, 690 ppm H_2_S	[29]
3	CO_2_ stream from the Cansolv^®^ absorption system	2.9% SO_2_	[30], as reported in a previous IEA GHG report
4	Predicted CO_2_ stream from a pre-combustion capture plant	1 vol% H_2_, 0.9 vol% N_2_, 300 ppm Ar, 100 ppm H_2_S, COS and other impurities	Composition data provided by IEA GHG [30]
5	Predicted CO_2_ stream from an oxy-fuel combustion plant	5.8 vol% N_2_, 4.7 vol% O_2_, 4.47 vol% Ar, 100 ppm NO_x_, 50 ppm SO_2_, 20 ppm SO_2_, 50 ppm CO	Composition data provided by IEA GHG [30]

**Table 4:** Summary of CO_2_ corrosion rates with different water concentrations from the literature (adapted by [32]).

N°	P (bar)	T (°C)	H_2_O (ppmv)	Material	t (h)	Flow	Corrosion Rate (mm/year)	Refs.
1	80	40	244	C-steel	168	Static	0.08	[72]
11	80	50	Sat	X65	14, 24, 48	Static	0.024 ≈ 0.1	[71]
26	79.6–82	35	10 g	SS: 304 L, 3161 C-Steel: X42, X60	120	100 rpm	SS: 0.0005–0.0008 C Steel: 0.007	[61]
29	80	50	650 (Undersat)	X65	24	Static	No corrosion	[64]
30	80	50	3310 (Sat)	X65	24	Static	0.38	[64,65,76]
31	80	50	10 g (Sat.)	X65	24	Static	0.4–1	[65]
32	75.8	40	244	C Steel	5	Static	1.2	[73,74,77]
35	79	31	244	C steel	5	Static	1.1	[73]
37	95 182	50–130	100 g (Sat.)	C steel	96	995	0.014–0.043	[63]
39	100	20	1220	X65	720	Static	No corrosion	[78]
45	100	25	488 e 1222	X65	336	3 rpm	0	[67]
46	125	80	1.5 g	38Mn6/C75	96	995	0.0036	[63]
49	123–146	25–60	Saturated	X65	48–400	180 rpm	0.01–0.1	[75]
50	240	50	40 g (Sat.)		24	Static	Not given	[56]

**Table 5:** Comparison of corrosion rates from literature under s-CO_2_ and water-saturated conditions.

Environment	Corrosion Rate (mm/years)	Refs.
H_2_O-rich phases	19.2	[63,64,65,75]
	10.6	[75]
	5–15	[32]
	0.6	[78]
	1.7	[78]
	5–30	[81,82]
CO_2_-rich phases	0.38	[75]
	0.013–0.043	[32]
	0.04	[78]
	0.01–0.1	[81,82]

**Table 6:** Comparison of the effect of O_2_ on the corrosion rate of steels in s-CO_2_.

N°	P	T	H_2_O	O_2_	Steel	t	Flow	Corrosion Rate	Refs.
	(bar)	(°C)	(ppmv)	(ppmv)		(h)	(rpm)	(mm/anno)	
1	75.8	40	2440	100		5	Static		[74]
2	80	50	Saturated (10 g)	0	X65	24	static	0.38	[65]
				2% (1.6 bar)				0.6	
				4% (3.3 bar)				1	
				6% (5.1 bar)				0.9	
3	80	50	650	3.3 bar	X65	24	Static	NO corrosion	[64]
			2000	4%				NO corrosion	
			3000					≤0.01	
4	79.6–82	35	Saturated (100 g)	0	304 L	120	100	0.002	[61]
					316 L			0.001	
					X42			0.014	
					X60				
5	94.8–103	49	Saturated (100 g)	3 v%	304 L	120	100	0.003	[61]
					316 L			0.004	
					X42			0.099	
					X60			0.093	
6	100	60	Saturated	Yes	X42	120	Static	0.008	[89]
			(1 mL 55.6 mmol)	(−1000 ppm)				(−1 mg) No corrosion	[89]
7	100	20	1220	488	x65	720	Static	No corrosion	[70]
8	100	10	50 v%	0	X65	312	Static	0.5	[70]
			20 (Saturated)			0.336		0.8	
			50	0		336		0.5	
			50	0		336		2.7	
			10	200		312		1.2	
			20	100		336		1.3	
			50	200		432		0.6 (pit corrosion rate 17)	
9	150	80	Saturated	1000 ppm		288	120	0.2–0.9	[90]
10	150	100	5% NaCl	0.045 bar	Type 420	720	static	0.08 Localized	[88]
				0.45 bar				0.25 Localized	
11	300	100	5% NaCl	0.045 bar	Type 420	720	static	0.07 Negligible localized	[88]
				0.45 bar				0.34 Localized	
12	150	100	5% NaCl	0.0045 bar	M-SS	720	Static	0.01 localized	[88]
				0.45 bar				0.04 Localized	
13	300	100	5% NaCl	0.045 bar	M-SS	720	Statie	0.02 No Localized	[88]
				0.45 bar				0.07 localized	
14	100		12	1000	X65	48–96	0–3 m/s	5 to10	[70]
			13	0				3 to 5	
15	80	50	400 ml Water phase	4%; 0	X65	24	Static	19.3; 19.2	[76]
						120		14.1; 10.6	
16	80		50,400 mL Water phase	4%	3Cr		Static	0.01	[64,65]

## Data Availability

No new data were created or analyzed in this study. Data sharing is not applicable to this article.
